# Phylogeographic and phylogenetic analysis for *Tripterygium* species delimitation

**DOI:** 10.1002/ece3.3344

**Published:** 2017-09-22

**Authors:** Baowei Ma, Tianyuan Hu, Pei Li, Qingjun Yuan, Zhaoshou Lin, Yuhe Tu, Jia Li, Xianan Zhang, Xiaoyi Wu, Xiujuan Wang, Luqi Huang, Wei Gao

**Affiliations:** ^1^ School of Traditional Chinese Medicine Capital Medical University Beijing China; ^2^ State Key Laboratory Breeding Base of Dao‐di Herbs National Resource Center for Chinese Materia Medica China Academy of Chinese Medical Sciences Beijing China; ^3^ Datian Taoyuan State Forest Farm in Fujian Province Datian China; ^4^ Yongan State Forest Farm in Fujian Province Yongan China

**Keywords:** haplotype network, phylogenetic analysis, phylogeographic analysis, species delimitation, *Tripterygium*

## Abstract

*Tripterygium wilfordii* (Celastraceae) is a traditional Chinese medicine; and the dried root and rhizome constitute the main officinal parts. *Tripterygium wilfordii* has been identified as a potential candidate for the treatment of systemic lupus erythematosus, rheumatoid arthritis, nephritis, asthma, leprosy, and cancer. The phylogenetic relationships within the *Tripterygium* genus are ambiguous; thus, our aim is to clarify the relationships within this genus using phylogeographic and phylogenetic analyses. Here, we first sequenced three plastid DNA regions (i.e., *psb*A*‐trn*H, *rpl32‐trn*L, and *trn*L‐*trn*F) and found that *Tripterygium hypoglaucum* and *T. wilfordii* were clustered together based on the strength of the topology in the phylogenetic analysis: *T*. *hypoglaucum* is polyphyletic, and *T. wilfordii* is paraphyletic. A spatial analysis of molecular variance showed that the best group value is 4, and the groups were almost consistent with the topology of in the phylogenetic analysis. The Mantel analyses of *Tripterygium* using IBD web showed statistically significant relationships between genetic and geographical distance distributions (*r* = .3479, *p* < .0001). The molecular dating using Fossil calibration indicated that the divergence in *Tripterygium* was approximately 8.13 Ma. Furthermore, we also analyzed four DNA regions (i.e., ITS2, *psb*A‐*trn*H, *mat*K, and *rbc*L) that were obtained from the NCBI nucleotide database; these results showed that *T. wilfordii* and *T. hypoglaucum* clustered together, while *Tripterygium regelii* represented a separate cluster. *Tripterygium hypoglaucum* and *T. wilfordii* were never distinct lineages, and the species circumscriptions are artificial. We propose that *T. wilfordii* and *T. hypoglaucum* are conspecific, while *T. regelii* likely constitutes a separate species.

## 1 INTRODUCTION

*Tripterygium wilfordii*, which is a perennial woody vine member of the Celastraceae family that is native to eastern and southern China, Taiwan, Korea, and Japan, has been used as a traditional and allopathic Chinese herb for centuries (Brinker, Ma, Lipsky, & Raskin, 2007). *Tripterygium wilfordii* extracts contain bioactive components, such as triptolide, celastrol, and tripchlorolide (Wong, Yuan, & Luk, 2012). Triptolide is a primary diterpene triepoxide that has been shown to have multiple important pharmacological effects, such as anti‐inflammatory activity and the inhibition of lymphocyte proliferation; Triptolide has also been used for the management of autoimmune diseases (Liu, 2011). Certain key enzyme genes in terpenoid biosynthesis in *T. wilfordii* Hook. f. such as 1‐deoxy‐d‐xylulose‐5‐phosphate synthase (DXS), 1‐deoxy‐d‐xylulose‐5‐phosphate reductoisomerase (DXR), farnesylpyrophosphate synthase, and 3‐hydroxy‐3‐methylglutaryl‐CoA synthase (HMGS) genes have been have been well studied (Liu et al., 2014; Tong et al., 2015; Zhao et al., 2015). Minnelide, which is a water‐soluble triptolide analog that is extracted from *Tripterygium*, has been shown to be highly effective in reducing pancreatic tumor growth and spread in mouse models (Chugh et al., 2012) and in suppressing small cell lung carcinoma tumor growth in animal models (Kumar, Corey, Scott, Shiva, & D'Cunha, 2016; Rousalova et al., 2013). (5R)‐5‐Hydroxytriptolide (LLDT‐8) is a new, optimized triptolide analog that shows lower cytotoxicity and relatively higher immunosuppressive activity (Shen et al., 2015). Moreover, triptonide can effectively inhibit canonical Wnt/β‐catenin signaling by targeting the downstream C‐terminal transcription domain of β‐catenin or a nuclear component associated with β‐catenin and inducing apoptosis in Wnt‐dependent cancer cells.

In addition to *T. wilfordii* Hook. f, there are other species in the *Tripterygium* genus, such as *Tripterygium bullockii* Hance, *Tripterygium forrestii* Loes, *Tripterygium hypoglaucum* (H. Lév.) Hutch, *Tripterygium regelii* Sprague & Takeda, and *Tripterygium doianum* Ohwi (Hance, 1880; Hutchinson, 1917; Ma, Brach, & Liu, 1999; Loesener & Theodor, 1913; Ohwi, 1932; Sprague & Takeda, 1912). The traditional morphological characteristics that are often used to separate the species within a genus are highly variable and cannot be used to separate these species; according to these characteristic, this genus comprises only one variable species (Ma et al., 1999). However, in the Germplasm Resource Information Network (https://www.arsgrin.gov/), *Tripterygium* contains the following four species: *T. wilfordii* Hook. f, *Tripterygium* spp, *T. hypoglaucum* (H. Lév.) Hutch, and *T. regelii* Sprague & Takeda. *T. hypoglaucum* (H. Lév.) Hutch shared a name with *Aspidopterys hypoglauca* H. Lév, *T. forrestii* Loes, and *T. wilfordii* var. *exesum* Sprague & Takeda (https://www.ars-grin.gov/). On the Tropicos site (http://www.tropicos.org/Home.aspx), *Tripterygium* contains the following five species: *T. bullockii* Hance, *T. forrestii* Loes, *T. hypoglaucum* (H. Lév.) Hutch, *T. regelii* Sprague & Takeda, and *T. wilfordii* Hook. f. *T. forrestii* var. *execum* (Sprague & Takeda) C.H. Wang was a variety of *T. forrestii* Loes. *T. wilfordii* var. *bullockii* (Hance) Matsuda, and *T. wilfordii* var. execum Sprague & Takeda were varieties of *T. wilfordii* Hook. f (http://www.tropicos.org/Home.aspx). In the Flora Republicae Popularis Sinicae (FRPS) publication, *Tripterygium* is considered to comprise the following three species: *T. wilfordii* Hook. f, *T. regelii* Sprague et Takeda, and *T. hypoglaucum* (Levl.) Hutch. *T. wilfordii* Hook. f shared a name with *T*. *bullockii* Hance and *T. wilfordii* Hook. f. var. bullockii (Hance) Matsuda. *T. wilfordii* auct. non Hook. f is the basionym of *T. regelii* Sprague et Takeda. *Aspidopteris hypoglaucum* Levl, *T. wilfordii* var. execum Sprague & Takeda, *T. forrestii* var. execum, and *T. forrestii* A. C. Smith are basionyms of *T. hypoglaucum* (Levl.) Hutch. However, the three species in the *Tripterygium* genus were named *T. wilfordii* Hook. f in the subsequent publication “Flora of China.” Unifying the species is often practical when addressing the problem of species delimitation (Wiens, 2007); however, incorrect species delimitation may lead to serious problems in further studies, and could result in a waste of money and effort.

Morphological data are responsible for most of our knowledge regarding the phylogeny of life (Scotland, Olmstead, & Bennett, 2003). However, there are limitations to these type of data, such as phenotypic plasticity, genetic variability (de Boer, Ichim, & Newmaster, 2015), and discrimination among cryptic species (Packer, Gibbs, Sheffield, & Hanner, 2009). Recently, DNA sequences have played an important role in species identification. The method used to identify organisms based on their DNA sequences has been coined DNA barcoding by Hebert, Cywinska, Ball, and Waard (2003) and includes the mitochondrial gene cytochrome *c* oxidase I gene as a standard barcode that is applied to all animals. Although many researchers have attempted to establish a universal plant barcode, none of the available sequences could be used for all species. The Barcode of Life‐Plant Working Group has shown that the *mat*K + *rbc*L pair is a useful plant barcode, with a discriminatory efficiency of 72 % (Group, 2009).

A quantitative analysis of 19 compounds has shown that there is a significant difference in the compound content between *T. wilfordii* and *T. hypoglaucum* (Guo, Duan, Liu, Liu, & Li, 2014). However, a content analysis of triptolide in different populations and individuals of *Tripterygium* suggested that triptolide in *T*. *regelii* was very low, and there was no significant difference between *T. wilfordii* and *T. hypoglaucum* (Huang, Guo, & Si, 2005). Furthermore, Chen et al. (2017) have shown that there was a significant difference (*p* < .0001) among the contents of 11 chemical components in *T. hypoglaucum*,* T. wilfordii,* and *T*. *regelii*. Molecular analyses using ITS and 5S rDNA sequences have shown that *T. hypoglaucum* is not distinct from *T. wilfordii*, while *T*. *regelii* can be recognized as an independent species (Law et al., 2011). Phylogenetic analyses of the *Celastrus* genus have shown that, based on the sequences of two nuclear (ETS and ITS) and three plastid (*psb*A*‐trn*H, *rpl16* and *trn*L‐F) sequences, *Celastrus* and *Tripterygium* (*T. wilfordii* and *T*. *regelii*) form a maximally supported clade (Mu, Zhao, & Zhang, 2012). In addition, an RAPD marker (Liu, Guo, Huang, & Si, 2007) experiment was conducted to study the genetic relationships and diversity in the *Tripterygium* genus. These experiments showed that five *T*. *regelii* individuals gathered closely to form a single branch that was separated from the other populations, and that *T. wilfordii* and *T. hypoglaucum* could be combined into the same species. Further analyses using four DNA regions (i.e., ITS2, *mat*K, *rbc*L, and *psb*A*‐trn*H) for species identification in *Tripterygium* showed that *T*. *regelii* clustered alone, whereas *T. hypoglaucum* clustered with *T. wilfordii*, suggesting that *T. hypoglaucum* and *T. wilfordii* are potentially conspecific or that the limited barcode did not provide enough variation for their differentiation (Zhang et al., 2016).

Here, our purpose is to clarify the species delimitation in the *Tripterygium* genus. We sequenced and combined three plastid DNA regions (i.e., *psb*A‐t*rn*H, *rpl*32‐*trn*L, and *trn*L‐*trn*F) as the first matrix, which was used in the population genetic analysis, phylogeographic analysis, phylogenetic analysis, and divergence time estimates. The other four DNA sequences (i.e., ITS2, *psb*A‐*trn*H, *mat*K, and *rbc*L) were downloaded from the National Center for Biotechnology Information (NCBI) nucleotide database (https://www.ncbi.nlm.nih.gov/nuccore) and combined as the second matrix, which was used in the phylogenetic analysis, including Bayesian inference (BI), maximum likelihood (ML), and haplotype network construction. Plastid DNA fragments are usually inherited maternally in angiosperms; however, the nuclear rDNA internal transcribed spacer is derived from both parents (McCauley, Sundby, Bailey, & Welch, 2007). The results show that *T. wilfordii* and *T. hypoglaucum* could be considered the same species, which is distinct from *T. regelii*. A spatial analysis of molecular variance (SAMOVA) showed that the best group value in *Tripterygium* is 4, the group from the southwest contains all specimens from *T. wilfordii* and some specimens from *T. hypoglaucum*, and all specimens of *T. r*egelii converge into northeast group alone. The NST value (0.854) was significantly (*p* < .05) greater than the GST (0.561), suggesting that there was a significant phylogeographic structure in *Tripterygium*. The Mantel analyses at the genus level indicated statistically significant relationships in the combined cpDNA sequences (*r* = .3479, *p* < .0001) between the genetic and geographical distance distributions. The molecular dating using Fossil calibration estimated that the divergence in *Tripterygium* was approximately 8.13 Ma.

## 2 MATERIALS AND METHODS

### 2.1 Sample preparation

Leaf samples were collected from three species in the *Tripterygium* genus and the species *Celastrus orbiculatus*. First, we collected *T. wilfordii* seedlings from different places in southern China and planted the seedings in Datian (Fujian Province in China) for further applications. In all cases, fresh leaves were plucked for the DNA extraction and stored at −80°C in the laboratory. Finally, 120 samples (Table S1) were collected as follows: 10 *T. hypoglaucum* samples, 10 *T. regelii* samples, 94 *T. wilfordii* samples, and 6 *C. orbiculatus* samples. The ITS2, *psb*A*‐trn*H, *rbc*L, and *mat*K intergenic region sequences in *Tripterygium* and *C. orbiculatus* were downloaded from the NCBI nucleotide database (Table S2).

### 2.2 DNA extraction, PCR amplification, and sequencing

Genomic DNA was directly extracted from fresh blades using the Plant Genomic DNA Kit (TIANGEN Biotech, Beijing, China) according to the manufacturer's protocol. DNA was then dissolved in double distilled water and stored at −20°C. After a preliminary screening of many DNA regions, we chose the *psb*A*‐trn*H (Sang, Crawford, & Stuessy, 1997) *rpl*32*‐trn*L (Shaw, Lickey, Schilling, & Small, 2007), and *trn*L*‐trn*F (Taberlet, Gielly, Pautou, & Bouvet, 1991) intergenic spacers (Table 1) for the subsequent analyses because this regions had the most polymorphic sites.

**Table 1:** Plastid regions and sequences of primers used in this study

Region	Primer names	Primer sequences
*psb*A*‐trn*H	*Fwd PA*	GTTATGCATGAACGTAATGCTC
	*Rev TH*	CGCGCATGGTGGATTCACAATCC
*rpl32‐trn*L	*trn*L^*(UAG)*^	CTGCTTCCTAAGAGCAGCGT
	*rpL32‐F*	CAGTTCCAAAAAAACGTACTTC
*trn*L*‐trn*F	*C*	CGAAATCGGTAGACGCTACG
	*F*	ATTTGAACTGGTGACACGAG

We used 20 μl PCRs containing 10 μl of 2×KAPA HiFi HotStart ReadyMix, 0.3 μmol/l of each primer, and 0.1–1 ng of DNA. PCR amplification was performed using a Veriti 96‐well thermal cycler (Applied Biosystems) that was programmed for an initial 3‐min step, followed by 35 cycles of 20 s at 98°C, 15 s at 56°C, 30 s (*psb*A*‐trn*H, *rpl32‐trn*L) or 50 s (*trn*L*‐trn*F) at 72°C, and a final 7 min step at 72°C. The PCR products were detected by electrophoresis on a 1% agarose gel containing GeneGreen Nucleic acid dye in TAE buffer; the fragments were visualized using the Vilber Lourmat imaging system. All PCR products were sequenced in both directions using an ABI 3730XL automated sequencer (Applied Biosystems) at the Beijing RUIBO Biotech Company.

### 2.3 Data analysis

#### 2.3.1 Sequences

The forward and reverse trace files that resulted from the sequencing were trimmed, visually inspected and manually adjusted using SeqMan version 7.1.0 (Burland, 2000). We removed the poor quality bases that were located at the 5′ and 3′ ends of the three types of PCR products. The individual alignments of the three cpDNA regions were then combined to produce a three‐gene alignment for all 120 samples using Editseq version 7.1.0 (DNASTAR, Madison, WI). A multiple sequence alignment was performed using MEGA version 7.0.18 (Kumar, Stecher, & Tamura, 2016). Haplotypes whose sites with gaps or missing were considered, invariable sites were included, and parsimony informative sites and invariable sites were analyzed using DNASP version 5 (Librado & Rozas, 2009). We also calculated haplotype diversity (*H*_d_) in *Tripterygium* using DNASP version 5 (Librado & Rozas, 2009). For the haplotype data set, the net between‐group mean distance that was used in the *p*‐distance model was calculated using MEGA version 7.0.18 (Kumar, Stecher, et al., 2016). In addition, Network version 5.0.0.0 (Fluxus Technology Ltd. 1999–2016) was used to construct a haplotype network based on the Median Joining (MJ) algorithm (Bandelt, Forster, & Röhl, 1999).

The *Tripterygium* and *C*. *orbiculatus* ITS2, *psb*A*‐trn*H, *matK,* and *rb*cL sequences were aligned using MEGA version 7.0.18 (Kumar, Stecher, et al., 2016) and then combined. The haplotype distribution was assessed using DNASP version 5 (Librado & Rozas, 2009), and a haplotype network based on the MJ algorithm (Bandelt et al., 1999) was analyzed using Network version 5.0.0.0 (Fluxus Technology Ltd. 1999–2016).

#### 2.3.2 Population genetic and phylogeographic analyses of *psb*A*‐trn*H +* rpl32‐trn*L +* trn*L*‐trn*F

The average within‐population diversity (HS), total gene diversity (HT), geographical total haplotype diversity (VT), geographical average haplotype diversity (VS), and differentiation among the populations (GST and NST) in *Tripterygium* were estimated based on the method proposed by Pons and Petit (Pons & Petit, 1995, 1996) using PermutCpSSR (http://www.pierroton.inra.fr/genetics/labo/Software). GST was dependent only on the haplotype frequencies, while NST also depends on the haplotype frequencies between the haplotypes. To identify possible population groups that are geographically homogeneous and maximally differentiated in 40 different locations in *Tripterygium*, a spatial analysis of the molecular variance (SAMOVA) was carried out using SAMOVA 2.0 (Dupanloup, Schneider, & Excoffier, 2002). The molecular distance was set to the pairwise difference, and the number of initial configuration groups was set to 100 with 1,000 simulated annealing steps. To select the most appropriate number of groups, the *K*‐values ranged from 2 to 35. While the rate of FCT change began to decline between successive *K*‐values, the best grouping of populations was determined (Blair et al., 2013). Analyses of molecular variance (AMOVA) (Excoffier, Smouse, & Quattro, 1992) about genetic structure of the populations were performed in order to analysis value of variation within populations (FST), among populations within groups (FSC), and among groups (FCT).

We estimated the associations between a genetic matrix based on the group average distance, which was calculated using MEGA7.0 with 1,000 bootstrap and a *p*‐distance model about of 40 locations in *Tripterygium*, and a matrix of the geographical distance (km) based on Mantel's (1967) method using IBDWS (http://ibdws.sdsu.edu/~ibdws/distances.html) (Jensen, Bohonak, & Kelley, 2005) with 10,000 random permutations.

### 2.4 Phylogenetic analysis and divergence time estimates

The BI and ML methods were used for constructing phylogenetic trees based on haplotype data sets of the combined cpDNA sequences. For the BI analysis, the model selection was based on the Akaike information criterion (AIC) using the program MrModelTest version 2.3 (Nylander, 2004). BI was analyzed using MrBayes version 3.2.6 (Huelsenbeck & Ronquist, 2001; Ronquist & Huelsenbeck, 2003; Ronquist et al., 2012) with *C. orbiculatus* as the outgroup. Two independent Bayesian Markov chain Monte Carlo (MCMC) analyses were conducted simultaneously. In these analysis, we used 2 × 10^6^ generations, sampled every 100 generations, and 25% (=5,000) of the trees were discarded as burn in with four MCMC chains using three heated chains and a cold chain. The verified convergence was shown in MrBayes and the average standard deviation of the split frequencies below 0.05. We set the Temp parameter as 0.05 so that we could control the heating coefficient. For the ML analysis, we implemented an automatic model selection (Lefort, Longueville, & Gascuel, 2017) with AIC using a Subtree Pruning and Regrafting ML heuristic with 1,000 bootstrap replications using PhyML version 3.0 (Guindon et al., 2010). The phylogenetic analysis of all *psb*A*‐trn*H +* rpl32‐trn*L +* trn*L*‐trn*F sequence combinations and the ITS2 + *psb*A‐*trn*H + *mat*K + *rbc*L sequence combinations is shown in Supplementary 3.

We estimated the time of the most recent common ancestor (TMRCA) of the haplotypes (*psb*A*‐trn*H +* rpl32‐trn*L +* trn*L*‐trn*F) using Beast version 2.3 (Drummond, Suchard, Xie, & Rambaut, 2012). The best substitution model was similar to that in the BI phylogenetic analysis. Fossils of *Tripterygium kabutoiwanum* were collected from the Japanese island, which dates to 5.332–11.608 Ma (http://fossilworks.org/bridge.pl?a=taxonInfo&taxon_name=Tripterygium). To calibrate the cpDNA substitution rate, the 27 Ma (Bacon, Simmons, Archer, Zhao, & Andriantiana, 2016) for the split between *Celastrus* and *Tripterygium* is set to Normal, and the 5.46–11.6 Ma for the split within *Tripterygium* is set to Log Normal. The Yule speciation hypothesis and uncorrelated relaxed lognormal molecular clock model were tested in Beast. MCMC chains were run for 10,000,000 generations and sampled every 1,000 generations. We used TRACER version 1.6 (Rambaut, Suchard, Xie, & Drummond, 2015) to test the results in Beast, and when the ESS values >200, the mixing and sampling are effective. The annotation of the final tree was executed using TreeAnnotator version 2.3. Figtree version 1.4.0 (http://tree.bio.ed.ac.uk/) was used to view the divergence times and topology.

## 3 RESULTS

### 3.1 Specimens and sequence analysis

**Figure 1:**
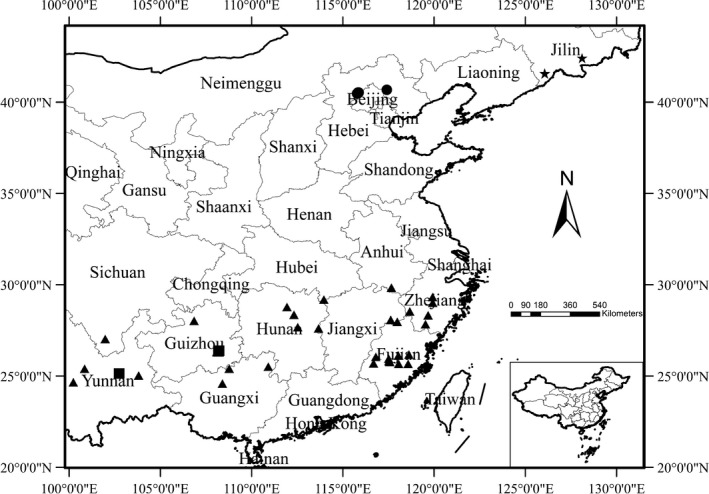
Geographical distribution of 120 samples. Maps were drawn using the software ArcGIS version 10.2 (http://desktop.arcgis.com). ▲: *Tripterygium wilfordii*; ■: *Tripterygium hypoglaucum*; ★: *Tripterygium regelii*; ●: *Celastrus orbiculatus*

One hundred and twenty samples were collected from 12 provinces in China (the geographical distribution is shown in Figure 1), and three cpDNA regions (*psb*A‐t*rn*H, *rpl*32‐*trn*L, and *trn*L‐*trn*F) were sequenced completely. We observed considerable length variation in the *psb*A*‐trn*H and *rpl32‐trn*L sequences in *Tripterygium*, which varied between 374‐399 and 544–562 bp. The *trn*L*‐trn*F alignment was 820 bp. The aligned matrix of *psb*A*‐trn*H was 400 characters in length with 13 parsimony informative sites, whereas the length of the *rpl32‐trn*L matrix was 570 characters with 11 parsimony informative sites, and the length of the *trn*L*‐trn*F aligned matrix was 820 characters with five parsimony informative sites. The total length of the concatenated alignment is 1,817 positions in 120 sequences. We observed 128 sites with alignment gaps or missing data, 1,614 invariable sites, and 75 variable (polymorphic) sites. The haplotype diversity (*H*_d_) is 0.7862 in *Tripterygium*. The net between‐group mean distance average using the *p*‐distance model in 18 (cHap1‐cHap18) haplotypes *C. orbiculatus—T. wilfordii* was 0.024496, *C. orbiculatus—T. hypoglaucum* was 0.024556, *C. orbiculatus—T. regelii* was 0.023373, *T. wilfordii —T. regelii* was 0.004127, *T. wilfordii—T. hypoglaucum* was 0.001942, and *T. regelii—T. hypoglaucum* was 0.004734.

Among the sequences downloaded from the NCBI nucleotide database, we obtained an ITS2 alignment of 223 bp with 10 parsimony informative sites; length variation was also observed in the *psb*A‐*trn*H sequence, which varied by 415–440 bp; and the aligned matrix was 440 characters in length with nine parsimony informative sites. The *rbc*L alignment was 502 bp with four parsimony informative sites, and the *mat*K alignment was 735 bp with one parsimony informative site. The total length of the concatenated alignment is 1,914 positions, the number of sites with alignment gaps or missing data is 44, the number of invariable sites is 1,814, and the number of variable (polymorphic) sites is 56. The haplotype diversity (*H*_d_) is 0.933 in *Tripterygium*. The net between‐group mean distance average using the *p*‐distance model in 15 (zHap1‐zHap18) haplotypes *C. orbiculatus*—*T. wilfordii* was 0.020856, *C. orbiculatus—T. hypoglaucum* was 0.021794, *C. orbiculatus—T. regelii* was 0.020856, *T. wilfordii—T. regelii* was 0.003922, *T. wilfordii — T. hypoglaucum* was 0.001687, and *T. regelii— T. hypoglaucum* was 0.005645.

### 3.2 Haplotype distribution and network

**Figure 2:**
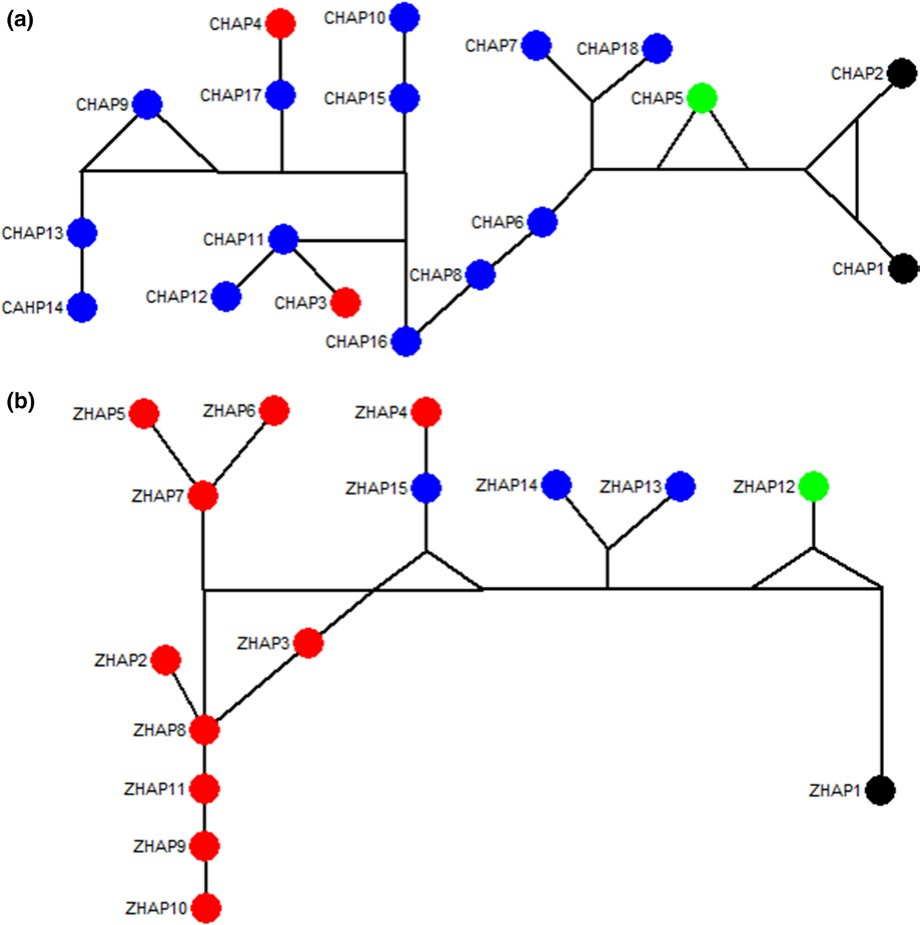
(a) Haplotype network of the combined three cpDNA (*psb*A‐t*rn*H, *rpl*32‐*trn*L, and *trn*L‐*trn*F); (b) Haplotype network of the combined four DNA (ITS2, *psb*A‐*trn*H, *mat*K, and *rbc*L) regions using median joining method. Blue dots stand for *Tripterygium wilfordii*, red dots stand for *Tripterygium hypoglaucum*, green dots stands for *Tripterygium regelii*, and black dots stand for *Celastrus orbiculatus*

The haplotype distribution was surveyed across the 120 individuals sequenced in this study. Eighteen haplotypes (cHap1‐cHap18) resulted from the combined cpDNA matrix (Table 2). Fifteen haplotypes (zHap1‐zHap15) resulted from the combined DNA (ITS2, *psb*A*‐trn*H, *mat*K, and *rb*cL) matrix (Table 3). The haplotype networks are shown in Figure 2. These haplotype networks revealed a direct connection between *T. wilfordii* and *T. hypoglaucum*, whereas *T. regelii* constitutes a distinct isolate.

**Table 2:** Detail message about the composition of haplotypes by the combined three cpDNA (*psb*A*‐trn*H +* rpl32‐trn*L +* trn*L*‐trn*F) regions

Name of haplotypes	Number of samples	Composition of samples
cHap1	5	CoBJ1 CoBJ2 CoBJ3 CoBJ4 CoBJ5
cHap2	1	CoBJ6
cHap3	5	ThGZ1 ThGZ2 ThGZ3 ThGZ4 ThGZ5
cHap4	5	ThYN1 ThYN2 ThYN3 ThYN4 ThYN5
cHap5	10	TrJL1 TrJL10 TrJL2 TrJL3 TrJL4 TrJL5 TrJL6 TrJL7 TrJL8 TrJL9
cHap6	49	TwAH1 TwAH2 TwAH3 TwAH4 TwAH5 TwAH6 TwAH7 TwAH8 TwFJ1 TwFJ10 TwFJ11 TwFJ12 TwFJ13 TwFJ14 TwFJ15 TwFJ16 TwFJ17 TwFJ18 TwFJ19 TwFJ2 TwFJ20 TwFJ21 TwFJ3 TwFJ5 TwFJ6 TwFJ7 TwFJ8 TwFJ9 TwGX1 TwGZ2 TwGZ3 TwGZ7 TwHB1 TwHB2 TwHB3 TwHB4 TwHB5 TwHB6 TwHB7 TwHB8 TwJX10 TwJX11 TwJX7 TwJX8 TwJX9 TwZJ2 TwZJ4 TwZJ5 TwZJ6
cHap7	1	TwFJ4
cHap8	1	TwGX2
cHap9	3	TwGX3 TwGX4 TwGX6
cHap10	1	TwGX5
cHap11	3	TwGZ1 TwGZ4 TwGZ5
cHap12	2	TwGZ6 TwGZ8
cHap13	13	TwHN1 TwHN10 TwHN11 TwHN2 TwHN3 TwHN4 TwHN5 TwHN6 TwHN7 TwHN8 TwHN9 TwZJ7 TwZJ8
cHap14	6	TwJX1 TwJX2 TwJX3 TwJX4 TwJX5 TwJX6
cHap15	6	TwSC1 TwSC2 TwSC3 TwSC4 TwSC6 TwZJ3
cHap16	1	TwSC5
cHap17	7	TwYN1 TwYN2 TwYN3 TwYN4 TwYN5 TwYN6 TwYN7
cHap18	1	TwZJ1

**Table 3:** Detail message about the composition of haplotypes by the combined four DNA (ITS2 + *psb*A‐*trn*H + *mat*K + *rbc*L) regions

Name of haplotypes	Number of samples	Composition of samples
zHap1	1	Co
zHap2	2	Th1 Th2
zHap3	1	Th10
zHap4	1	Th11
zHap5	1	Th12
zHap6	2	Th13 Th14
zHap7	1	Th15
zHap8	1	Th3
zHap9	4	Th4 Th5 Th6 Th7
zHap10	1	Th8
zHap11	1	Th9
zHap12	5	Tr1 Tr2 Tr3 Tr4 Tr5
zHap13	1	Tw1
zHap14	2	Tw2 Tw3
zHap15	2	Tw4 Tw5

### 3.3 Population genetic and phylogeographic analyses

The PermutCpSSR results for *psb*A*‐trn*H +* rpl32‐trn*L +* trn*L*‐trn*F revealed a significant phylogeographic structure in *Tripterygium* because the GST value (0.561) was significantly (*p* < .05) smaller than the NST value (0.854). The total gene diversity (HT = 0.981) is much higher than the average within‐population diversity (HS = 0.430); the value of the geographical total haplotype diversity (VT) and geographical average haplotype diversity (VS) was 1.069 and 0.156, respectively. The result of the SAMOVA of the multiple *K*‐values evaluated for cpDNA sequences is shown in Table 4. The results imply that the FCT values did not have the highest value; however, when *K* > 8, at least one of the groups contained a single population, suggesting that the group structure was disappearing (Heuertz et al., 2004). When the *K* value is 4 (Table 4), the rate of change in the FCT values began to decline. The Mantel analysis of all populations of *Tripterygium* was conducted on the IBD website by comparing the genetic and geographical distance distributions and showed statistically significant relationships in the combined cpDNA sequences (*r* = .3479, *p* < .0001). However, when the four groups were analyzed separately, no statistically significant relationship was observed in either group (*p* > .05).

**Table 4:** Results of spatial analysis of the molecular variance (SAMOVA) for *K* = 2–7

*K* value	FSC	FST	FCT (Rate of change value)	Groups *K* = 4
2	0.82353	0.94151	0.66854	*SOUTHWEST*: ThGZ1 ThGZ2 ThGZ3 ThGZ4 ThGZ5 ThYN1 ThYN2 ThYN3 ThYN4 ThYN5 TwGZ1 TwGZ2 TwGZ3 TwGZ4 TwGZ5 TwGZ6 TwGZ7 TwGZ8 TwSC1 TwSC2 TwSC3 TwSC4 TwSC5 TwSC6 TwYN1 TwYN2 TwYN3 TwYN4 TwYN5 TwYN6 TwYN7 *NORTHEAST*: TrJL1 TrJL2 TrJL3 TrJL4 TrJL5 TrJL6 TrJL7 TrJL8 TrJL9 TrJL10 *SOUTHEAST*: TwAH1 TwAH2 TwAH3 TwAH4 TwAH5 TwAH6 TwAH7 TwAH8 TwFJ1 TwFJ10 TwFJ11 TwFJ12 TwFJ13 TwFJ14 TwFJ15 TwFJ16 TwFJ17 TwFJ18 TwFJ19 TwFJ20 TwFJ21 TwFJ2 TwFJ3 TwFJ4 TwFJ5 TwFJ6 TwFJ7 TwFJ8 TwFJ9 TwGX1 TwGX2 TwHB1 TwHB2 TwHB3 TwHB4 TwHB5 TwHB6 TwHB7 TwHB8 TwJX7 TwJX8 TwJX9 TwJX10 TwJX11 TwZJ1 TwZJ2 TwZJ4 TwZJ5 TwZJ6 *SOUTH*: TwGX3 TwGX4 TwGX5 TwGX6 TwHN1 TwHN2 TwHN3 TwHN4 TwHN5 TwHN6 TwHN7 TwHN8 TwHN9 TwHN10 TwHN11 TwJX1 TwJX2 TwJX3 TwJX4 TwJX5 TwJX6 TwZJ3 TwZJ7 TwZJ8
3	0.77325	0.93615	0.71841 (0.04987)	
4	0.68183	0.93303	0.78951 (0.07110)	
5	0.58853	0.93148	0.83347 (0.04396)	
6	0.53193	0.93132	0.85328 (0.01981)	
7	0.49528	0.93071	0.86271 (0.00943)	

The all *p*‐values of FCT, FST, and FSC were estimated based on 1,000 random initial conditions, and exhibited high significance (*p* < .001) in each tested group.

### 3.4 Phylogenetic analysis and divergence time estimates

**Figure 3:**
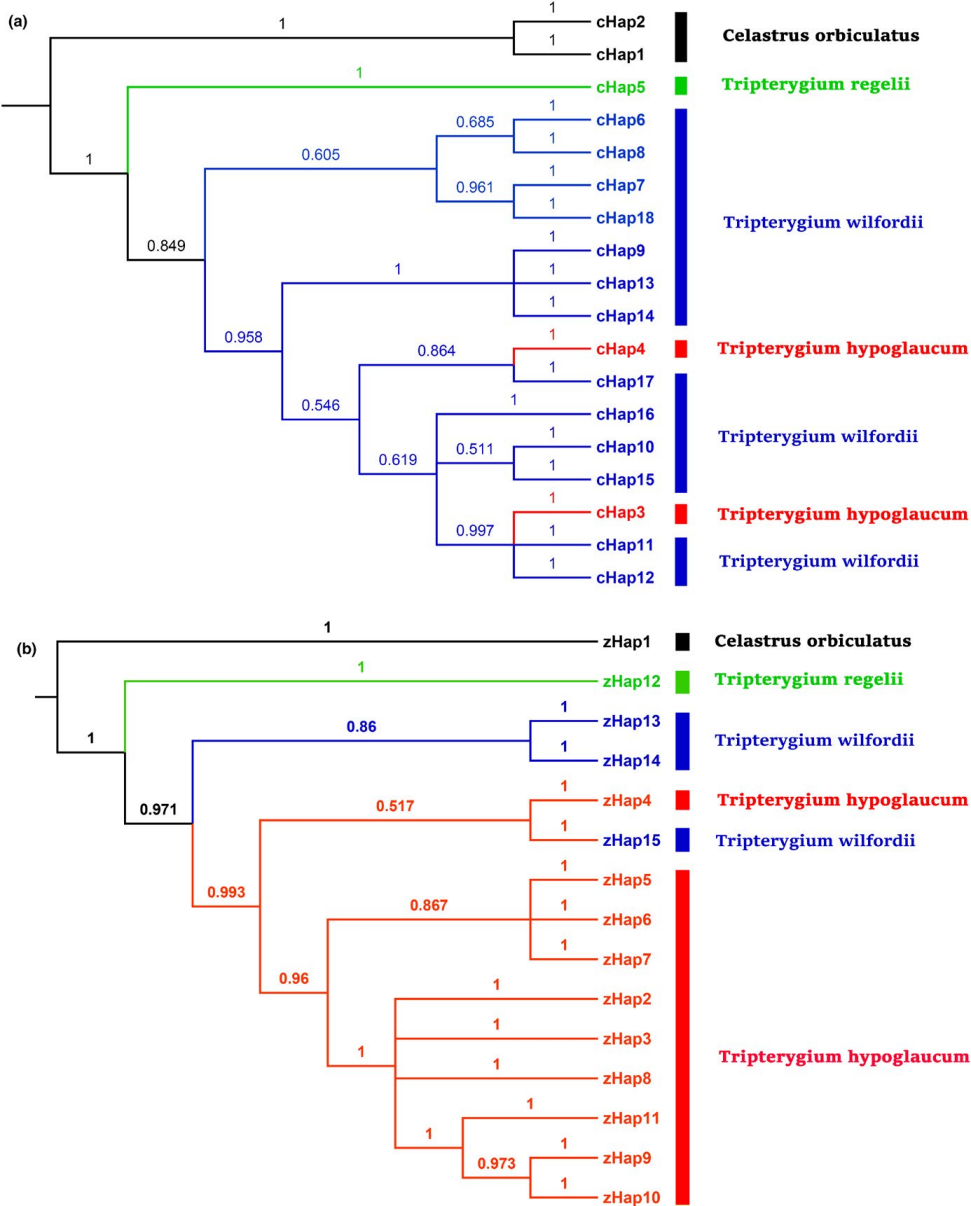
Phylogenetic trees of haplotypes based on the combined three plastid DNA regions (a) (*psb*A‐t*rn*H, *rpl*32‐*trn*L, and *trn*L‐*trn*F) and the combined four DNA regions (b) (ITS2, *psb*A‐*trn*H, *mat*K, and *rbc*L) using Bayesian inference method. The number on each branch indicates the posterior probability (PP). Model selection: (a) GTR+I+G; (b) GTR+I. The average standard deviation of the split frequencies, (a) 0.006417; (b) 0.002667

The BI haplotype phylogenetic trees were based on the *psb*A*‐trn*H +* rpl32‐trn*L +* trn*L*‐trn*F regions and the combination of the ITS2 + *psb*A‐*trn*H + *mat*K + *rbc*L regions and are presented in Figure 3. The haplotype topologies of the rooted trees constructed using the BI and ML methods were nearly identical same, except for a more evident explicit internal evolutionary relationship that is evident in the ML trees (Supplementary 3). All trees that were constructed using both methods showed that *C. orbiculatus* and *T. regelii* were distinct species. In contrast, the haplotypes of *T. hypoglaucum* and *T. wilfordii* were not clustered independently and often overlapped with each other. We also found that there was a basal split between two sister groups in the *Tripterygium* genus.

**Figure 4:**
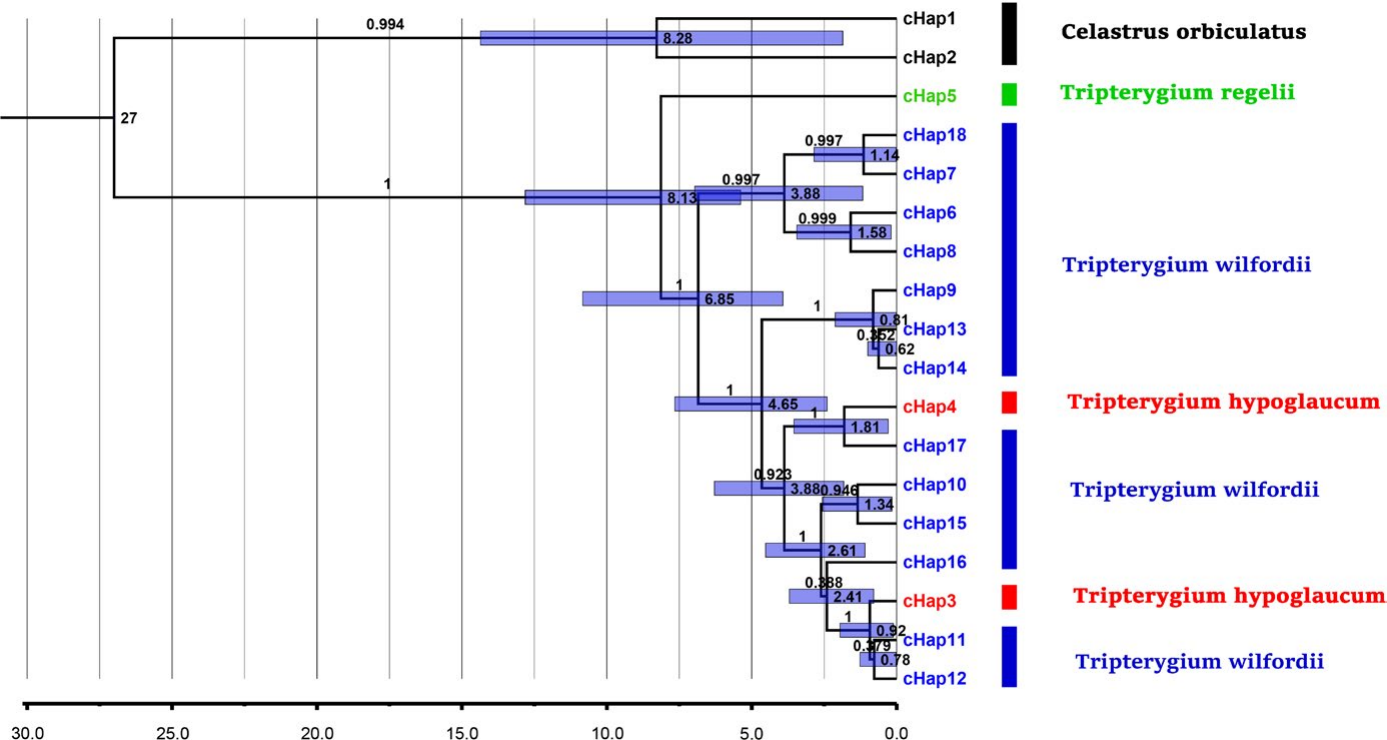
BEAST‐derived chronogram of haplotypes based on the combined cpDNA (*psb*A‐t*rn*H, *rpl*32‐*trn*L, and *trn*L‐*trn*F) sequences. Blue bars on each node show 95% highest posterior density (HPD) confidence intervals for divergence estimates. The number on each branch indicates the posterior probability (PP). The node age (Myr) of the major lineages was shown near the blue bars

The molecular dating using Fossil calibration estimated the divergence in *Tripterygium* (Figure 4) at approximately 8.13 Ma (lower 95 % Highest Posterior Density, HPD = 5.38 Ma, upper 95 % HPD = 12.81 Ma), suggesting that the original *Tripterygium* occurred during the Miocene epoch. The TMRCA of *T. wilfordii* and *T. hypoglaucum* is estimated at 6.85 Ma (95 % HPD, 3.92 Ma–10.83 Ma). The TMRCA of clades cHap3 and cHap4 is 0.92 Ma (95 % HPD, 0.12 Ma–1.95 Ma) and 1.81 Ma (95 % HPD, 0.29 Ma–3.55 Ma), respectively.

## 4 DISCUSSION

*Tripterygium* is an annual woody vine member of the Celastraceae family and is at mostly 1–4 m. During our sampling, we found that most of the morphological characteristics are variable and have alternative characteristics states in species of *Tripterygium*. The petiole is mostly polygonal and is 0.5–2 cm long. The leaf blade is usually ovate or rounded‐ovate, oblong or elliptic‐ovate and is 10–15 cm long and 4–9 cm wide; occasionally, there is white powder on the lower leaf surface in *T. hypoglaucum* and *T. wilfordii*. Inflorescence is thyrse that is composed of several to dozens of thyrselets. The flowers are whitish, and the petals are oblong to subovate. However, morphological characteristics are not sufficient to properly assign the species status to *T. wilfordii* or *T. hypoglaucum*. The presence of white powder on the lower leaf surface has traditionally been the main character used for identifying *T. wilfordii* and *T. hypoglaucum*. In previous observations, certain specimens had white powder (although its presence varied depending on the location and habitat), certain specimens lacked white powder, and certain specimens inconspicuous powder (Ma et al., 1999). In addition, the size and shape of the leaves, inflorescences, and fruit were variable depending on the habitat and time of collection (Fitter & Hay, 1987). The pubescence growing on the surface of the branches and branchlets was also variable depending on the specific parts and growth period in *T. wilfordii*, making it difficult to use this character to divide the genus.

Every species spans a certain geographical range, and the ecological and evolutionary processes that limit the geographical range may be crucial for creating new species, particularly allopatric species (Wiens, 2007). *Tripterygium wilfordii* Hook. f. is native to Taiwan, Fujian, Jiangsu, Zhejiang, Anhui, Hubei, Hunan, and Guangxi provinces; *T. hypoglaucum* (Levl.) Hutch is native to the Anhui, Zhejiang, Hunan, Guangxi, Guizhou, Yunnan, and Sichuan provinces; and *T. regelii* Sprague et Takeda is native to the Jilin and Liaoning provinces. *Tripterygium wilfordii* and *T. regelii* are geographically isolated, and phylogenetic analyses have shown that these species are likely different species; however, there is no geographical isolation between *T. wilfordii* and *T. hypoglaucum*. Moreover, our results showed that the net between‐group mean distance used the *p*‐distance model about *T. wilfordii—T. regelii* and *T. regelii*—*T. hypoglaucum* is greater than the distance in *T. wilfordii—T. hypoglaucum*.

High levels of genetic diversity (HT = 0.981) were detected in the *Tripterygium* combined cpDNA using PermutCpSSR. Narrowly distributed species may have lower levels of genetic diversity than widespread species (Cole, 2003; Hamrick & Godt, 1996). The wide distribution of *Tripterygium* in south and northeast China potentially lead to a high level of genetic diversity. We found that the significant phylogeographic structure of *Tripterygium* was related to the significantly greater NST values compared with the GST value (NST = 0.854 > GST = 0.561; *p* < .05). The four genetically differentiated population groups of *Tripterygium*, which are located in northeast, southeast, southwest, and south China, were determined by SAMOVA. In the southwest group determined by SAMOVA, the samples of all *T. hypoglaucum* and certain *T. wilfordii* cluster together; the northeast group contained *T. regelii*; and the southeast and southern groups contained *T. wilfordii*. In addition, the groups almost corresponded to the topology of phylogenetic analysis. The Mantel analyses of all populations of *Tripterygium* showed a statistically significant relationship (*r* = .3479, *p* < .0001) between the genetic and geographical distances in the combined cpDNA sequences.

China has a long history and a rich culture of herbal medicines, and medicinal plants account for 11,146 species from 2,309 genera and 383 families (Chen et al., 2010). In the herbal products market, herbal medicinal materials are often substituted by herbs from related species or are contaminated with unlabeled fillers (Ming, Cao, But, & Shaw, 2011). DNA barcoding may, thus, provide a feasible and cost‐effective tool for species authentication and the monitoring of the herbal products. DNA barcoding can potentially be used to determine the identity of plant the species used in herbal medicines that may cause adverse drug reactions, although none of the available loci apply to all species (Li et al., 2015). To conduct the phylogeographic and phylogenetic analyses for the *Tripterygium* species delimitation, we combined the following three cpDNA regions: *psb*A*‐trn*H, *rpl32‐trn*L, and *trn*L*‐trn*F. Currently, *psb*A*‐trn*H is widely used for DNA barcoding due to its highly conserved coding sequence (which facilitates primer design) (Shaw et al., 2005); *psb*A*‐trn*H can be easily amplified across a broad range of nearly all angiosperms (Shaw et al., 2007). The *psb*A*‐trn*H cpDNA intergenic region exhibits a large number of insertions/deletions (Kress & Erickson, 2007). The *rpl32‐trn*L intergenic spacer, which is located in a small single‐copy region of the chloroplast genome, has also been used in sequence‐based phylogenetic analyses of *Cistus creticus* (Falchi et al., 2009), the Antirrhineae family (Yousefi, Zarre, & Heubl, 2016), the *Ombrocharis* genus (Chen et al., 2016), and the American Vernonieae tribe (Loeuille, Keeley, & Pirani, 2015). *Trn*L*‐trn*F is also a chloroplast spacer that has been frequently used for studying phylogenetic relationships and species identification (Shaw et al., 2007).

More than 22 concepts of species are in use today but many of them are notably inconsistent in terms of biological diversity (Mayden, 1997). Delimitating species based on morphological or breeding data is often problematic, and population genetics and phylogenetics should be ideally incorporated. According to the phylogenetic species concept, species that survived natural selection and descent can be identified based on their reciprocal monophyly and/or diagnosability (Leliaert et al., 2014; Mayden, 1997). In the lineage species concept, species can diverge from each other due to multiple mechanisms, such as a disrupted gene flow, local adaptation, intrinsic reproductive isolation, and introgression (De Queiroz, 2007). The concept of separately evolving meta population lineages has been argued and accepted by several authors (De Queiroz, 2007; Mayden, 1997). Gene trees based on homologous genes or DNA regions spanning intraspecific and interspecific evolution are vital for the rapid and cost‐effective understanding of the process of speciation (Templeton, 2001). Gene trees are often implicitly equated to species trees, and notably, the branches of a species tree imply population genetic processes (Leliaert et al., 2014; Maddison, 1997). Because gene trees contain large amounts of significant information regarding the speciation process, molecular data (e.g., the commonly used DNA fragments) are vital for species identification, species discovery, and species delimitation (Wiens, 2007). Hall (2005) analyzed several phylogenetic methods that use protein and DNA sequences and found that BI trees that are based on DNA sequences had been aligned according to the alignment of the corresponding protein sequences were the most accurate, followed by ML trees based on DNA sequences and parsimony trees based on protein sequences. Here, we used the ML and BI methods based on DNA sequences to carefully evaluate the partitioning of the *Tripterygium* genus. The combination of *psbA‐trn*H + *rpl32‐trn*L + *trnL‐trn*F and ITS2 + *psbA‐trn*H + *mat*K + *rbc*L based phylogenetic trees of haplotypes constructed using the BI and ML methods showed that *Tripterygium wilfordii* and *Tripterygium hypoglaucum* were conspecific. This observation appears to concur with Mu et al. (Mu et al., 2012) and Sue Ka‐Yee Law's (Law et al., 2011) recognition of *T*. *regelii* as a distinct species from *T. wilfordii* and *T. hypoglaucum*. The divergence time estimates show that the divergence of all *Tripterygium* combined cpDNA haplotypes (8.13 Ma; 95 % HPD, 5.38 Ma–12.81 Ma) most likely began during the Miocene epoch, and *Tripterygium* survived the quaternary glaciation. The TMRCA of *T. hypoglaucum* began during the Pliocene and Pleistocene, which occurred after the TMRCA of the two major clusters of *T. wilfordii* (6.85 Ma; 95 % HPD, 3.92 Ma–10.83 Ma), which began during the Miocene. The topologies of the phylogenetic trees of all populations show a congruence with the result of the haplotype network.

In conclusion, the results of the phylogeographic and phylogenetic analyses of the cpDNA regions in the leaf from *Tripterygium* show that *T. hypoglaucum* and *T. wilfordii* were clustered together in the phylogenetic trees, haplotype network, and a SAMOVA; in addition, the net between‐group mean distance in *T. wilfordii*—*T*. *regelii* and *T*. *regelii*—*T. hypoglaucum* is greater than the distance in *T. wilfordii*—*T. hypoglaucum*. *T. wilfordii* and *T. hypoglaucum* could be considered a single species with the name *T. wilfordii* Hook. f, while *T*. *regelii* is a separate species with the name *T*. *regelii* Sprague et Takeda.

## CONFLICT OF INTEREST

None declared.

## AUTHOR CONTRIBUTIONS

W.G., L.Q.H. and B.W.M. conceived the study; B.W.M. conducted the experiment and wrote the manuscript; B.W.M., Y.H.T., P.L, Q.J.Y., Z.S.L., Y.H.T., L.J., X.N.Z., X.Y.W., and X.Y.W. collected specimens and performed data analysis. This work was supported by the National Natural Science Foundation of China (81422053 and 81373906 to W.G).

## DATA ACCESSIBILITY

The data sequenced in my study have been submitted to the Nucleotide of NCBI (KY323346–KY323705). Other sequences were downloaded from the NCBI nucleotide database (KP644425–KP644524, KJ716428, KF022392, LC006125, JQ424179)
