# Supplementary material for: Phylogeographic and phylogenetic analysis for Tripterygium species delimitation
Source: Ecol Evol. 2017 Sep 22;7(20):8612–23. doi: 10.1002/ece3.3344 (PMC5648662; doi:10.1002/ece3.3344)
Supplement: Supplementary file 1 [file ECE3-7-8612-s001.docx]

**Haplotype network and phylogenetic analysis for *Tripterygium* species** **delimitation**

BAOWEI MA^1^, TIANYUAN HU^1^, PEI LI^1^, QINGJUN YUAN^2^, ZHAOSHOU LIN^3^, YUHE TU^4^, JIA LI^1^, XIANAN ZHANG^1^, XIAOYI WU^1^, XIUJUAN WANG^1^,

LUQI HUANG^2^, WEI GAO^1*^

1. School of Traditional Chinese Medicine, Capital Medical University, Beijing, China

2. State Key Laboratory Breeding Base of Dao-di Herbs, National Resource Center for Chinese Materia Medica, China Academy of Chinese Medical Sciences, Beijing, China

3. Datian Taoyuan State Forest Farm in Fujian Province, Datian, China

4. Yongan State Forest Farm in Fujian Province, Yongan, China


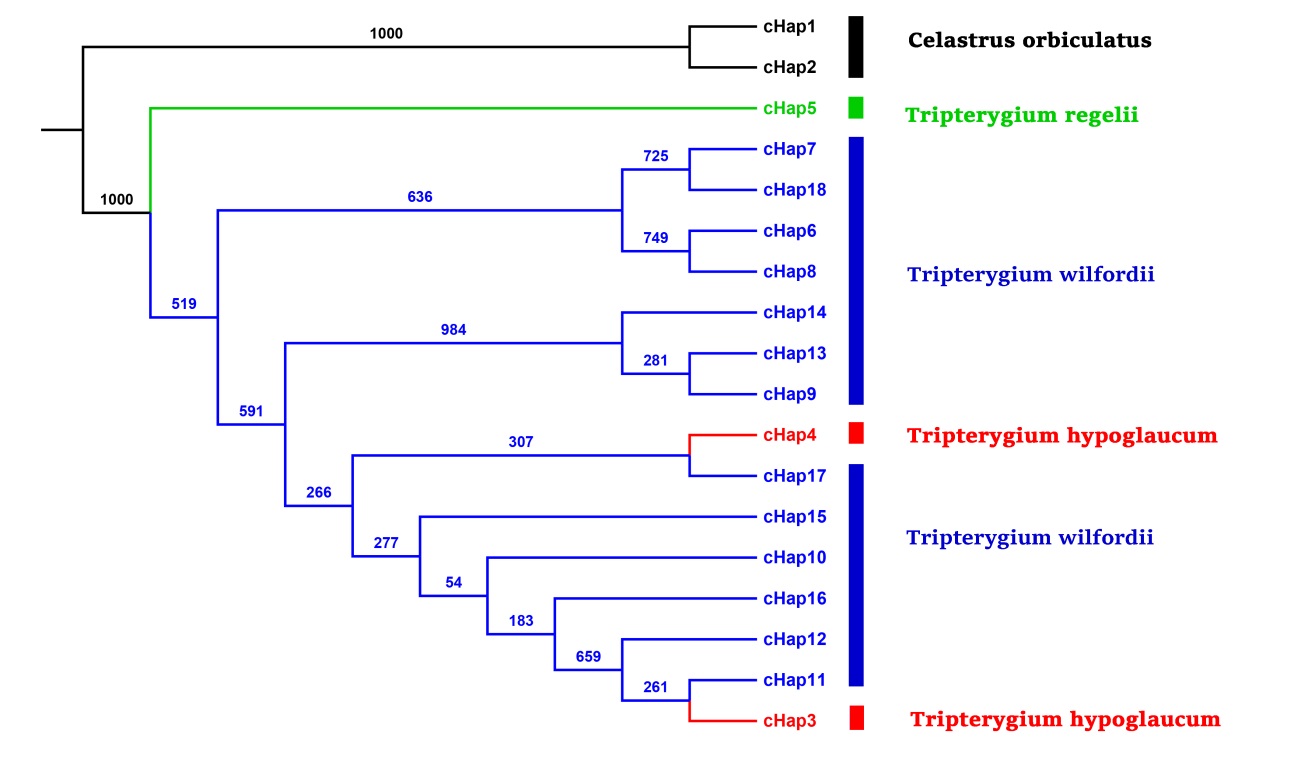


**Figure 1 Phylogenetic trees of haplotypes based on the combined three plastid DNA regions (*psb*A-t*rn*H, *rpl*32-*trn*L and *trn*L-*trn*F) using the Maximum Likelihood method** (Guindon et al., 2010)**.** The number on each branch indicates the bootstrap value (BS). Model selection: GTR+G+I.


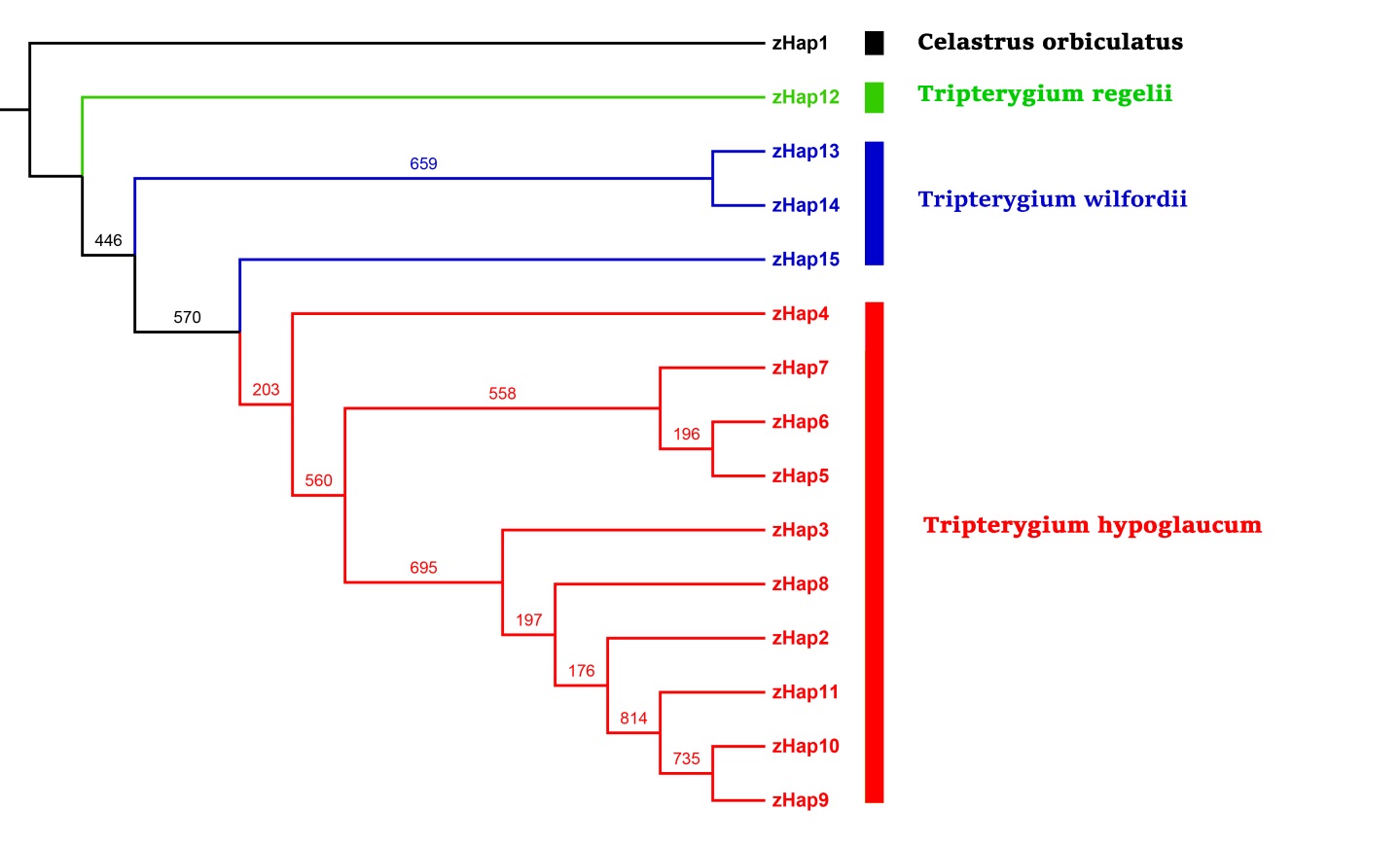


**Figure 2 Phylogenetic trees of haplotypes based on the combined four plastid DNA regions (ITS2, *psb*A-*trn*H, *mat*K and *rbc*L) using the Maximum Likelihood method** (Guindon et al., 2010)**.** The number on each branch indicates the bootstrap value (BS). Model selection: GTR+I.

**
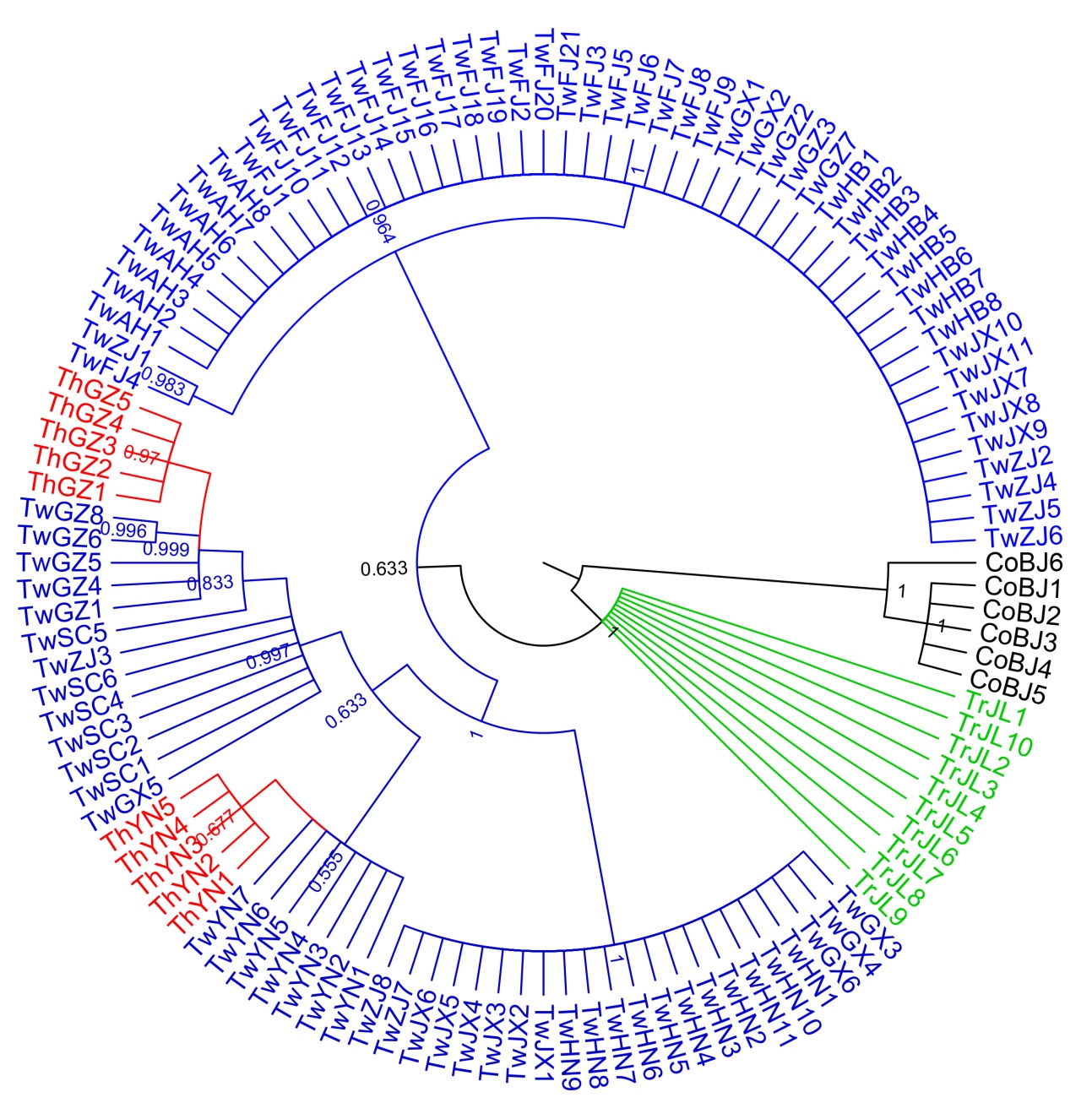
**

**Figure 3 Phylogenetic tree of combined three plastid DNA regions (*psb*A-t*rn*H, *rpl*32-*trn*L and *trn*L-*trn*F) by Bayesian Inference method** (Nylander, 2004;Huelsenbeck & Ronquist 2001; Ronquist & Huelsenbeck 2003; Ronquist *et al.* 2012)**.** Bayesian Inference was performed in mrbayes version 3.2.6, running GTR+I+G model for the combined three cpDNA regions with Celastrus orbiculatus as the outgroup. For the analysis we used 2 x10^6^ generations, sampled every 100 generations, twenty five percent (=5000) of the trees were discarded as burn in with four Markov chain Monte Carlo (MCMC). The number on each node indicates the posterior probability (PP). The average standard deviation of the split frequencies: 0.018383.


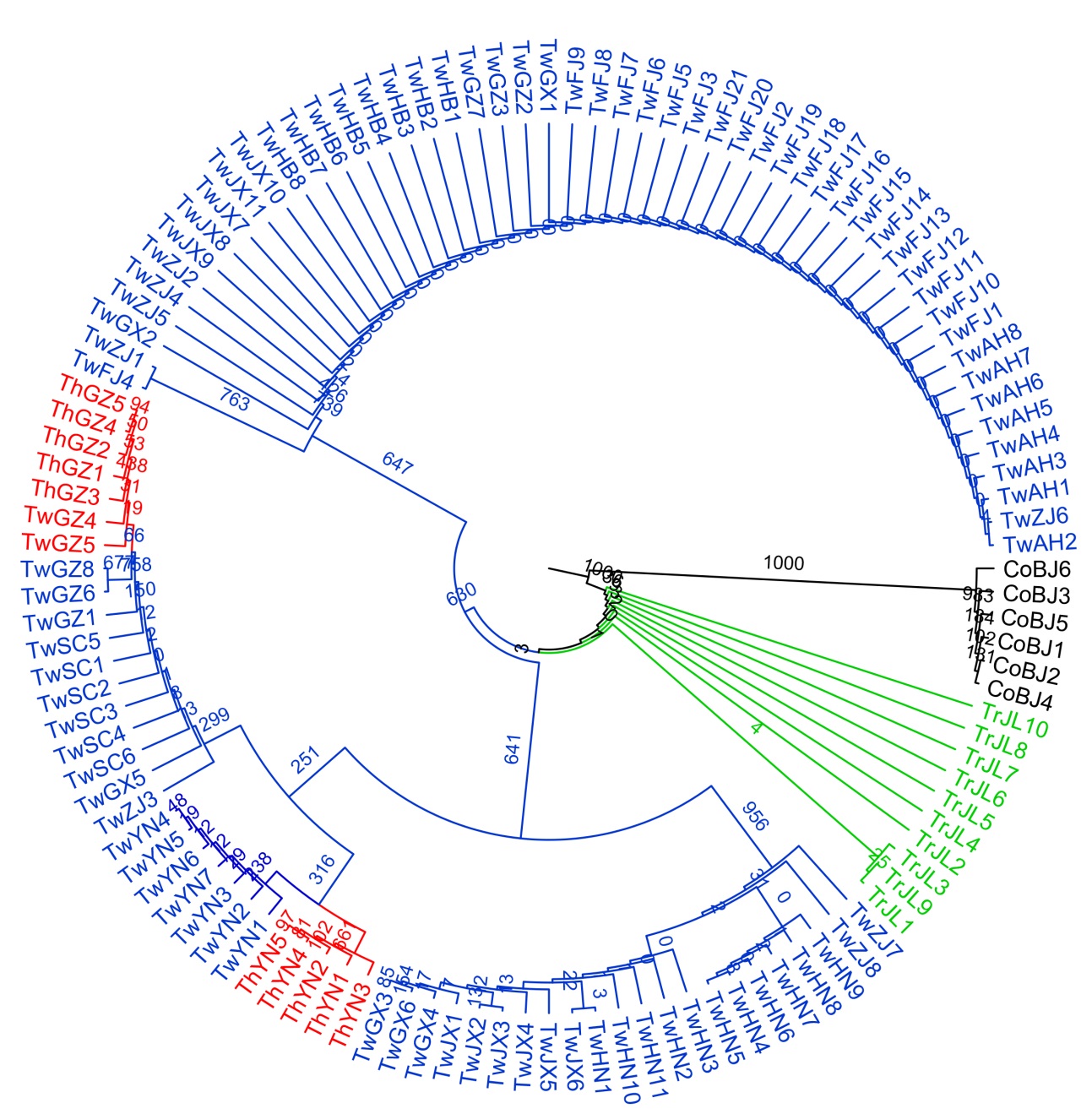


**Figure 4 Phylogenetic tree of combined three plastid DNA regions (*psb*A-t*rn*H, *rpl*32-*trn*L and *trn*L-*trn*F) by Maximum Likelihood method** (Guindon et al., 2010)**.** Automatic model selection with AIC in Maximum Likelihood analysis using GTR+G+I+F model for the combined three cpDNA regions using Subtree Pruning and Regrafting (SPR) ML heuristic with 1000 bootstrap replications were implemented in PhyML version 3.0.The number on each branch indicates the bootstrap value (BS).

**
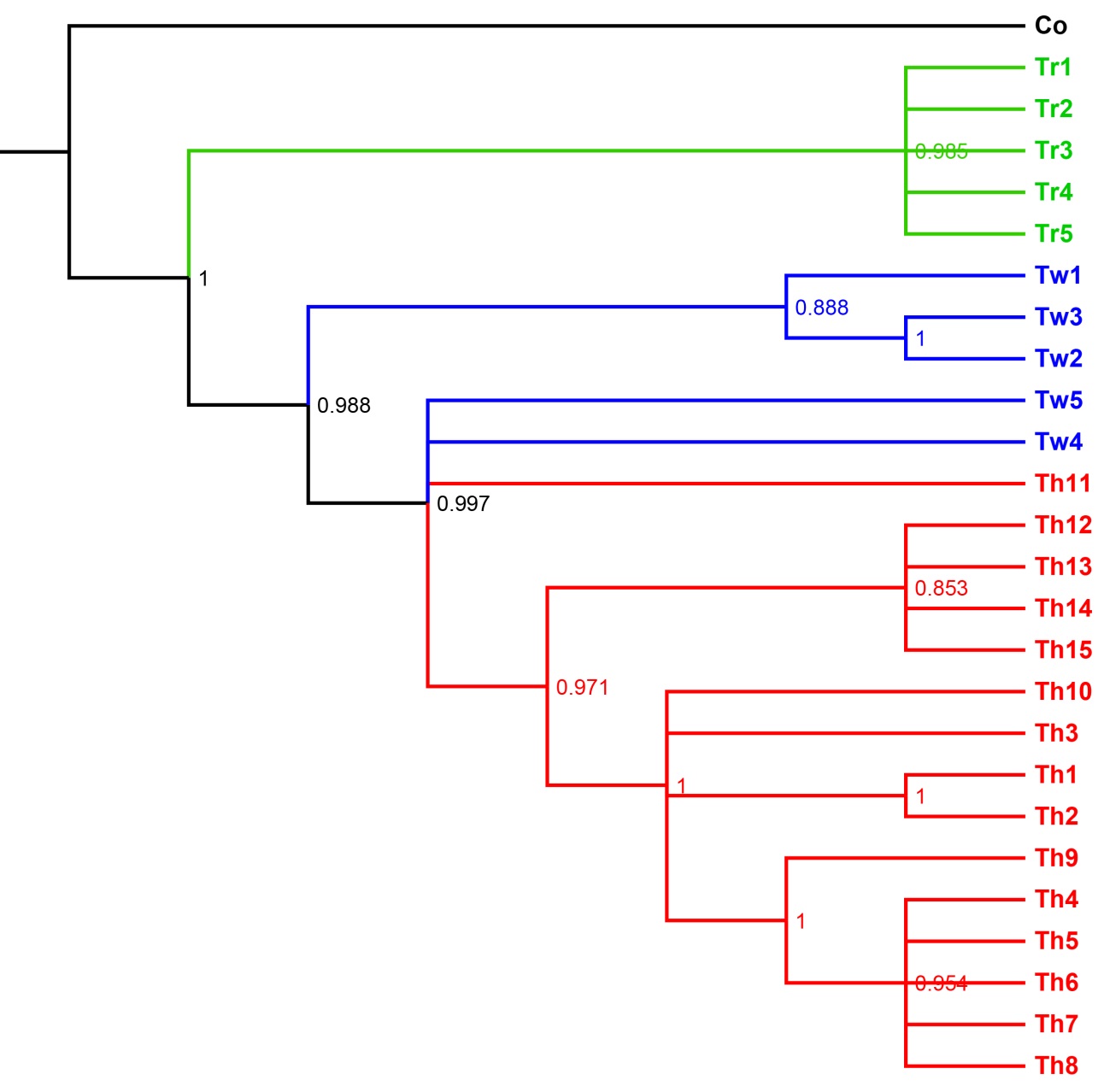
**

**Figure 5. Phylogenetic tree of the combined four DNA regions（ITS2+ *psb*A-*trn*H+ *mat*K+ *rbc*L） by Bayesian Inference method** (Nylander, 2004; Huelsenbeck & Ronquist 2001; Ronquist & Huelsenbeck 2003; Ronquist *et al.* 2012)**.** Bayesian Inference was performed in mrbayes version 3.2.6, running GTR+I+G model with *Celastrus orbiculatus* as the outgroup. For the analysis we used 2 x10^6^ generations, sampled every 100 generations, twenty five percent (=5000) of the trees were discarded as burn in with four Markov chain Monte Carlo (MCMC). The number on each node indicates the posterior probability (PP). The average standard deviation of the split frequencies: 0.003061.


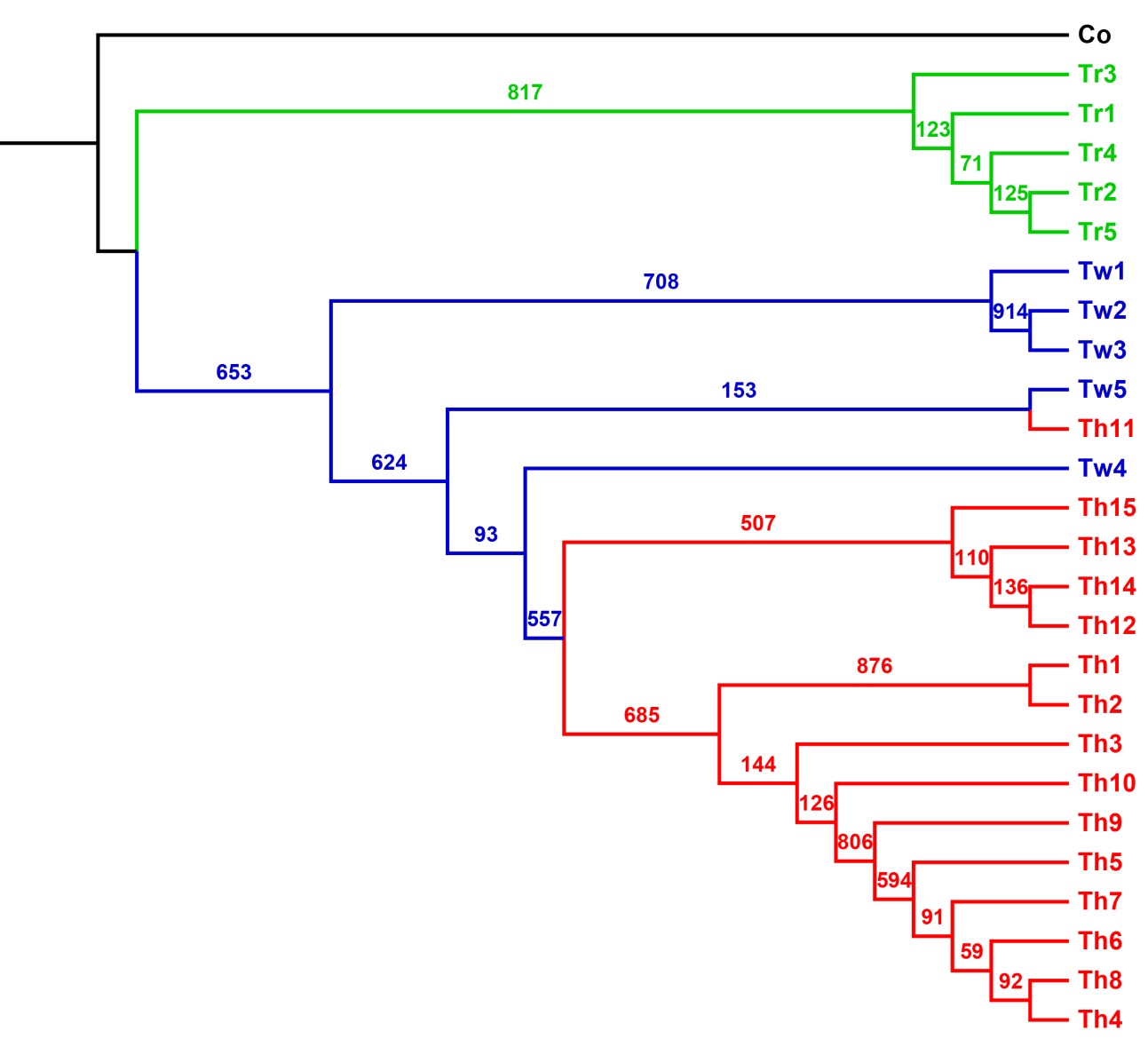


**Figure 6. Phylogenetic tree of the combined four DNA regions（ITS2+ *psb*A-*trn*H+ *mat*K+ *rbc*L） by Maximum Likelihood method** (Guindon et al., 2010)**.** Automatic model selection with AIC in Maximum Likelihood analysis using GTR+ I model using Subtree Pruning and Regrafting (SPR) heuristic with 1000 bootstrap replications were implemented in PhyML version 3.0.The number on each branch indicates the bootstrap value (BS).

Nylander, J. A. A. (2004) MrModeltest V2. Program Distributed by the Author. *Evolutionary Biology Centre* Uppsala University, 2, 1-2.

Guindon, S., Dufayard, J. F., Lefort, V., Anisimova, M., Hordijk, W., Gascuel, O. (2010). New algorithms and methods to estimate maximum-likelihood phylogenies: assessing the performance of PhyML 3.0. *Syst Biol* **59**, 307-321.

Huelsenbeck, J. P., & Ronquist, F. (2001). MRBAYES: Bayesian inference of phylogenetic trees. *Bioinformatics* **17**, 754-755.

Ronquist, F., & Huelsenbeck, J. P. (2003) MrBayes 3: Bayesian phylogenetic inference under mixed models. *Bioinformatics* **19**, 1572.

Ronquist, F., Teslenko, M., Van Der Mark, P., Ayres, D. L., Darling, A., Höhna, S., ... Huelsenbeck, J. P. (2012) MrBayes 3.2: efficient Bayesian phylogenetic inference and model choice across a large model space. *Systematic Biology* **61**, 539.
